# Capillary pumping independent of the liquid surface energy and viscosity

**DOI:** 10.1038/s41378-018-0002-9

**Published:** 2018-03-26

**Authors:** Weijin Guo, Jonas Hansson, Wouter van der Wijngaart

**Affiliations:** Micro and Nanosystems, KTH Royal Institute of Technology, Osquldas väg 10, Stockholm, 100 44 Sweden

## Abstract

Capillary pumping is an attractive means of liquid actuation because it is a passive mechanism, i.e., it does not rely on an external energy supply during operation. The capillary flow rate generally depends on the liquid sample viscosity and surface energy. This poses a problem for capillary-driven systems that rely on a predictable flow rate and for which the sample viscosity or surface energy are not precisely known. Here, we introduce the capillary pumping of sample liquids with a flow rate that is constant in time and independent of the sample viscosity and sample surface energy. These features are enabled by a design in which a well-characterized pump liquid is capillarily imbibed into the downstream section of the pump and thereby pulls the unknown sample liquid into the upstream pump section. The downstream pump geometry is designed to exert a Laplace pressure and fluidic resistance that are substantially larger than those exerted by the upstream pump geometry on the sample liquid. Hence, the influence of the unknown sample liquid on the flow rate is negligible. We experimentally tested pumps of the new design with a variety of sample liquids, including water, different samples of whole blood, different samples of urine, isopropanol, mineral oil, and glycerol. The capillary filling speeds of these liquids vary by more than a factor 1000 when imbibed to a standard constant cross-section glass capillary. In our new pump design, 20 filling tests involving these liquid samples with vastly different properties resulted in a constant volumetric flow rate in the range of 20.96–24.76 μL/min. We expect this novel capillary design to have immediate applications in lab-on-a-chip systems and diagnostic devices.

## Fluid Mechanics: Novel capillary pump design with constant flow

A new capillary pump design achieves constant flow independent of sample viscosity and surface energy. Capillary flow is a liquid transport phenomenon on the micro- and nanoscale and is used in a wide range of applications. Achieving precise control of flow rates is crucial to the function of many of those applications, especially diagnostic biochemical tests. Among the factors that limit the precision with such tests are variability in the surface energy and viscosity of the sample; a team headed by Wouter van der Wijngaart at the KTH Royal Institute of Technology, Micro and Nanosystems in Stockholm addressed those two factors. The team designed a capillary pump with constant flow that is unaffected by sample viscosity and surface energy. The authors believe their design will find immediate application with lab-on-a-chip systems and diagnostic devices.

## Introduction

As outlined in previous studies^1^, capillary flow is a dominant liquid transport phenomenon at the microscale and nanoscale. Capillary flow modification is used in many applications, e.g., to manipulate liquids in heat pipes^2^, to regulate fluid flow in low-gravity environments in space^3^, to pattern biomolecules on surfaces^4^, in the pumping mechanism of immunoassays^5^, and in diagnostic applications^6^. In many of these applications, precise control of flow rates is of central importance to their function. This is especially evident for diagnostic applications, such as immunoassays, where the biosensor signal depends on a biochemical reaction that is directly affected by the reaction time and by the transport of the sample within the specific area that this reaction occurs.^7^ Specifically, in lateral flow tests, the assay performance is typically limited to relatively high concentrations of the target molecule (high limit of detection) and to qualitative, and sometimes semi-quantitative, results. These limitations stem from various sources of variability, including (i) the geometry and surface chemistry of the porous capillary matrices; (ii) the variability of the surface energy and viscosity of the sample; and (iii) the variability in the diffusivity and binding constants of the analyte in the sample. Synthetic micropillar array capillary matrices address variability issues of the porous material.^8–10^ This work addresses the variability of the sample viscosity and surface energy.

Capillary flow rate linearly depends on the surface energy and inversely linearly depends on the viscosity, and variations in those liquid sample properties lead to the same relative variations in the flow rate. In biomedical applications, for example, the person-to-person variations in the fluidic properties of clinical samples of urine and whole blood from healthy individuals are 2.9 and 6.4% (standard deviation) for surface energy^11,12^ and 10% and 14% for viscosity^13,14^, respectively. Even larger deviations occur in many health conditions (e.g., samples of polycythaemia patients have ~4× larger viscosities than those of regular patients, and the blood viscosity of adults is considerably lower than that of infants^15^). The effect of these variations on diagnostic test performance is well recognized in the literature^16,17^, although it has not been quantitatively studied and is difficult to analytically predict.

Capillary flow and its control have been the subjects of extensive research since the beginning of the twentieth century. After a short initial acceleration phase, the flow in capillary pumps is defined by the capillary pressure drop over the liquid–gas interfaces and the viscous losses in the transported fluids^1,18^. In capillary devices with constant cross-sections, this leads to the well-known Washburn behavior, which is characterized by a flow rate that depends on the square root of time^19–21^. Constant capillary flow can be obtained by introducing a large upstream liquid resistance that dominates the viscous losses in the capillary device^22,23^. Sample viscosity-independent capillary pumping can be obtained by introducing a downstream fluidic resistance for the displaced air that dominates the viscous losses in the capillary system^1^. Sample surface energy-independent capillary flow has not yet been reported.

In this paper, we introduce and experimentally demonstrate the capillary pumping of a liquid sample with a flow rate constant in time and independent of the sample viscosity and surface energy.

**Fig. 1:**
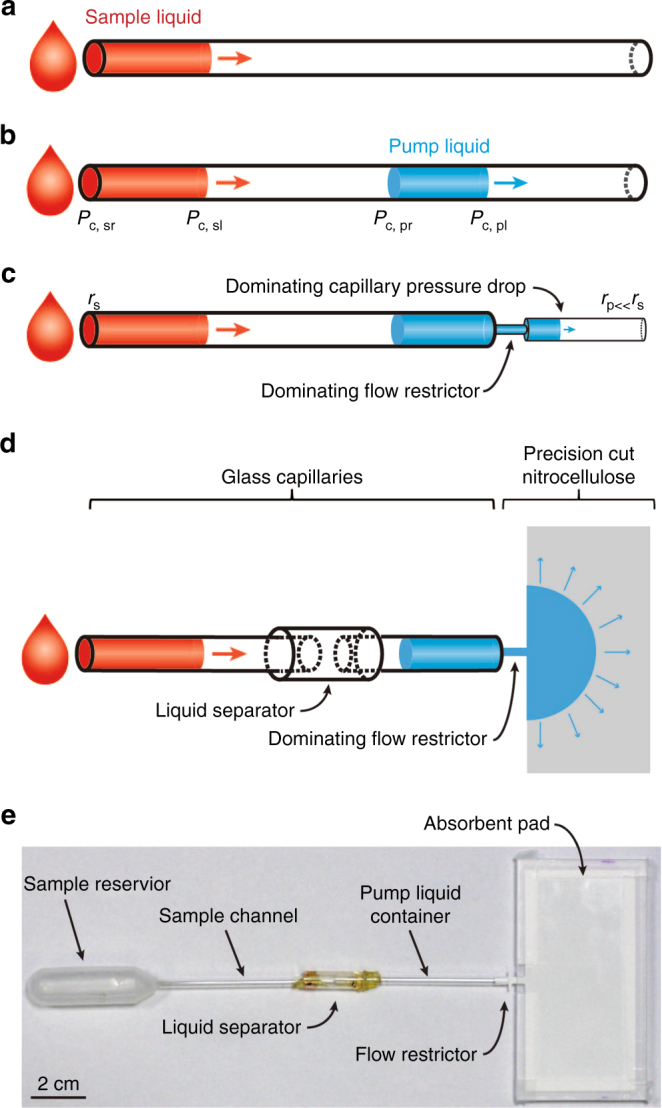
Design and implementation of the capillary pump principle. **a** The capillary flow of a sample liquid in a constant cross-section capillary depends on the sample liquid properties; **b** adding a second liquid, the pump liquid generates a flow dependency on both the sample liquid and the pump liquid; **c** creating a flow restrictor and decreasing the capillary cross-section in the pump liquid region generates a flow dependency solely on the pump liquid; **d** sketch of the capillary pump using three concatenated glass capillary tubes and precision cut nitrocellulose paper; and **e** photograph of a capillary pump.

We propose a capillary pump design that consists of an upstream sample section concatenated to a downstream pump section. The downstream pump section contains a well-characterized pump liquid plug that capillarily imbibes the pump and thereby pulls along the unknown sample liquid in the upstream sample section, as illustrated in Fig. 1. The flow rate is governed by the Laplace pressure drops over the four liquid–gas interfaces: the leading and rear front of the pump liquid plug and the leading and rear front of the sample liquid plug, indicated with indices pl, pr, sl, and sr, respectively. The pump is designed such that the net Laplace pressure over the pump liquid is much larger than that over the sample liquid, which is expressed as follows.

1 $${{P}_{\rm{c, pl}}}{\kern 1pt} {\mathrm{-}}{\kern 1pt} {{P}_{\rm{c, pr}}}{\kern 1pt} {\mathrm{\gg }}{\kern 1pt} {{P}_{\rm{c,sl}}}{\kern 1pt} {\mathrm{-}}{\kern 1pt} {{P}_{\rm{c,sr}}}.$$

Moreover, a flow restrictor in the pump section is designed to have a substantially higher fluidic resistance, R_R_, than the rest of the device. These design features ensure that the flow rate of the entire pump is dominated by the capillary pressure over and the viscous losses in the pump liquid plug. Hence, the flow rate is independent of the sample liquid properties. A constant capillary pressure drop, *P*_c,pl_ – *P*_c,pr_, over the pump liquid plug and a near constant fluidic resistance of the entire device, *R*_total_ ≈ R_R_, ensure a time-independent volumetric flow rate that linearly scales with the capillary cross-sectional size, *r*, which is expressed as follows.

2 $$Q \approx \frac{{P_{\rm{c,pl}}}}{{R_R}}\sim r$$

High flow rates can be obtained by upscaling the device; however, scaling is ultimately limited when the effects of gravity start dominating those of surface energy. In the Supplementary Information, we illustrate that volumetric flow rates exceeding 1 mL/s may be feasible with appropriate geometrical design.

## Materials and methods

We constructed capillary pumps, as shown in Fig. 1, in which we satisfy inequality 1 by choosing the following parameters,

3 $${P_{\rm{c,pl}}}{\kern 1pt} {\mathrm{\gg }}{\kern 1pt} {P_{\rm{c,pr}}}{\kern 1pt} {\mathrm{\approx }}{\kern 1pt} {P_{\rm{c,sl}}}{\kern 1pt} {\mathrm{\approx }}{\kern 1pt} {P_{\rm{c,sr}}}$$

and by choosing an absorbent pad section of which the internal flow resistance is *R*_P_*« R*_R_. The pump consists of two identical capillary glass tubes (Sigma-Aldrich, Switzerland) of length 62 mm, ID 2.5 mm and OD 3.3 mm for the pump liquid and sample liquid channels; a plastic capillary (cut from 1 ml syringe, Norm-Ject^®^, Henke-Sass Wolf, Germany) of length 21 mm, ID 4.7 mm, and OD 6.5 mm for the capillary valve; and a piece of nitrocellulose paper (Hi-Flow Plus HF075, Merck Millipore, Germany) with a manufacturer-specified capillary filling time *t*_fill_ = 75 ± 8 s (i.e., 10.7% variation) for a strip with length *l* = 4 cm. The strip is precision cut by a xurograph (Cutting Plotter CE5000-60, Graphtech). A precision machined PMMA (Poly(methyl methacrylate)) case is used to prevent uncontrolled evaporation from the nitrocellulose absorption pad.

First, the two thin capillaries are glued to the wide capillary using a thiol-ene-based UV-curing adhesive polymer (Ostemer 220, Mercene Labs, Sweden), such that the abrupt widening of the capillary system creates a geometric capillary valve^24^ that acts as a liquid separator for liquids entering from either side through the smaller capillaries and prevents cross reactions between the two. After capillary gluing, we primed the pump liquid channel with DI water as the pump liquid by capillary filling. The nitrocellulose paper was cut to contain, from upstream to downstream, a narrow insertion region that can be fitted into the pump liquid channel, a wider section that controls the insertion depth of the paper into the pump liquid channel during operation, a narrow section that forms the flow restrictor, and a wide section that forms the absorbent pad. The PMMA case consists of a flat roof and a grooved bottom sealed together with tape around the nitrocellulose absorbent pad.

We experimentally tested the following sample liquids with varying viscosity and surface energy properties: DI water (18 MΩ cm, Millipore), ethanol (99.5%, Analytical Grade, Solveco, Sweden), isopropanol (LC-MS Chromasolv, Sigma-Aldrich, Germany), mineral oil (Mineral oil, light, 330,779 Sigma-Aldrich, USA), glycerol (ACS reagent, ≥99.5%, Sigma-Aldrich, USA), urine from three different persons, and whole blood from three different persons (stored in EDTA tubes). The influence of the liquid properties on capillary imbibition can be expressed by a number *κ* that we define as follows:

4 $$\kappa = \frac{\mu }{{\gamma \cdot {\rm{cos}}(\theta )}},$$

where *μ* is the liquid viscosity, *γ* is the liquid–air surface energy, and *θ* is the contact angle between the liquid and the pump material. *κ*^−1^ is a measure of the capillary filling velocity of the liquid in a given capillary system. For all sample liquids tested, we determined *κ* using standard capillaries, i.e., capillary tubes with constant radius, *r*, and length, *l*, by measuring their filling time, *t*_fill_. The filling behavior of standard capillary tubes is described by the Washburn equation:

$$l^2 = \frac{{\gamma \cdot {\rm{cos}}(\theta ) \cdot r \cdot t_{\rm{fill}}}}{{2 \mu }}$$

which allows us to calculate *κ* from the following expression.

$$\kappa = \frac{{r \cdot t_{\rm{fill}}}}{{2 l^2}}$$

We measured the contact angles of the different sample liquids on a plasma-cleaned glass slide (25 mm × 75 mm, VWR, Germany) using a Theta Lite tensiometer (Biolin Scientific, Sweden).

To operate the device, we placed the sample liquid at the entrance of the sample liquid channel. The capillary filling process was initiated by inserting the nitrocellulose paper into the pump liquid channel. Capillary filling was recorded with a digital camera (Canon EOS 600D, Japan) and analyzed to determine the liquid front position over time.

The pump rate of all liquid samples was measured once, except for that of DI water, which was measured ten times. The environmental temperature and relative humidity were recorded at the beginning of every measurement.

To evaluate the characteristics of our novel design compared to those of the previously reported viscosity-independent capillary pump^1^, we also tested pumping DI water, ethanol, isopropanol, mineral oil, and glycerol using viscosity-independent pumps. The details and results of these measurements are provided in the Supplementary Information.

## Results

The measured sample liquid contact angles (on plasma-cleaned glass slides), values of *κ*, and average flow speeds for both the viscosity-independent pump design (previous design, see Supplementary Information) and the viscosity- and surface energy-independent pump design (this work), together with values of the viscosity^25–28^ and surface energy found in the literature, are listed in Table 1 for the common lab liquids tested. Table 2 lists the results for the clinical samples.

**Table 1:** Properties of lab and household liquids and their flow rates during capillary pumping

Sample liquid properties	DI water	Ethanol	Isopropanol	Mineral oil	Glycerol
Contact angle *θ*	0°	0°	0°	31.0°	13.1°
Viscosity *μ* (mPa s)	1.00^25^	1.07^26^	1.96^27^	68	1.412 × 10^3^^28^
Surface energy *γ* (mJ/m^2^)	71.60	20.01	18.86	28.27	89.8
*κ*	0.025	0.071	0.154	2.05	27.8
Viscosity-independent pump speed^a^	1	0.23	0.27	0.19	0.59
Viscosity- and surface energy-independent pump speed^a^	1	1.01	0.98	0.93	0.99

^a^The pump speed is normalized based on the average measured pump speed for DI water.

**Table 2:** Properties of clinical samples and their flow rates during capillary pumping

Sample liquid properties	Blood sample 1	Blood sample 2	Blood sample 3	Urine sample 1	Urine sample 2	Urine sample 3
Viscosity *μ* (mPa s)	3–4^14^			52.32–59.46^13^		
Surface energy *γ* (mJ/m^2^)	52.32–59.46^12^			70–72^11^		
Contact angle *θ*	15.6°	15.9°	16.5°	0°	0°	0°
*κ*	0.64	0.37	0.49	0.039	0.029	0.058
Viscosity- and surface energy-independent pump speed^a^	1.08	1.04	0.99	1.04	1.06	0.92

^a^The pump speed is normalized based on the average measured pump speed for DI water.

The values of *κ* for the sample liquids vary between 0.025 and 27.8 s/m, i.e., by three orders of magnitude. This variation stems from differences in both the surface energy and viscosity of the liquid samples.

**Fig. 2:**
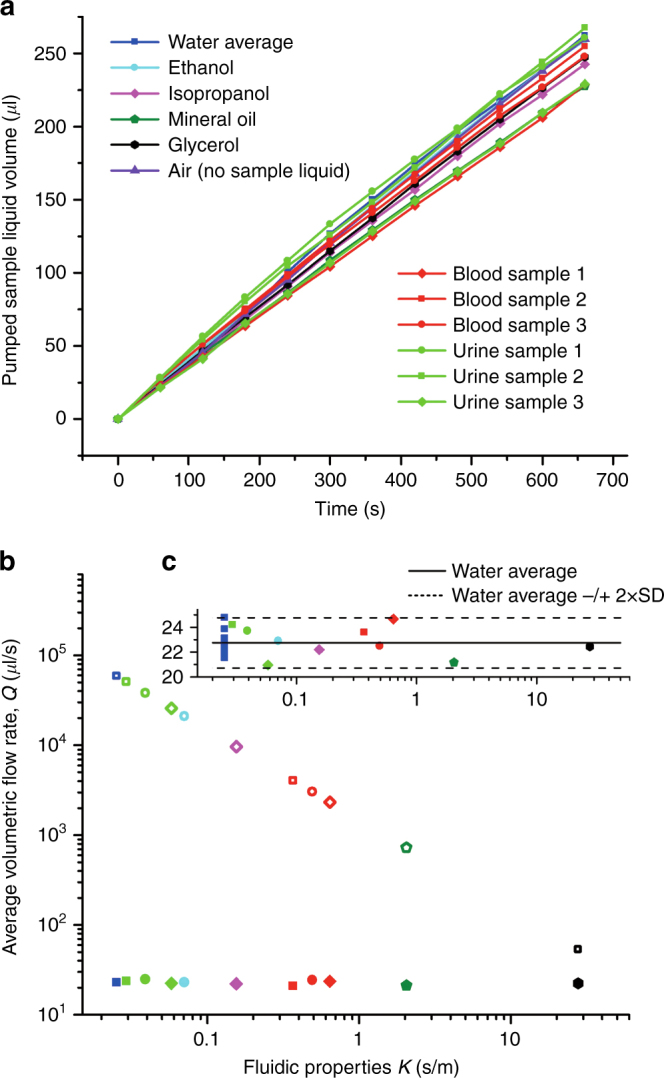
**a** Measurements of the pumped sample volume versus time for our novel capillary pump. Solid lines are for visualization purposes only. **b** Capillary volumetric flow rate, *Q*, versus *κ* for the sample liquids tested in our novel capillary pump (solid markers) and in standard capillaries (hollow markers). The volumetric flow rate was determined as the ratio of the measured total pumped sample volume over the measured total pumping time. The value depicted for water is the average over ten measurements. **c** Zoomed view of the novel capillary pump flow rate results with the ten flow rate values for water plotted separately. The solid and dashed lines indicate the average flow rate for water and two times the standard deviation of the flow rate for water, respectively.

Figure 2a shows a plot of the imbibing sample liquid volumes versus time for the novel pump design. The relation between the pumped volume and time is linear, indicating a constant flow rate. A plot of the imbibing sample liquid volumes versus time for the viscosity-independent pump is shown in the Supplementary Information.

Figure 2b shows a plot of the average volumetric flow rate, *Q*, versus liquid properties, *κ*, for the filling of standard capillaries and for the new pump design. Standard capillaries provide a flow rate of *Q* ~ *κ*^−1^, whereas the new pump design provides a constant flow rate of *Q*_av_ = 22.85 ± 1.11 (SD) μL/min ~ *κ*^0^. The ten measurements of water imbibition exhibited a standard deviation of *σ*_*ω*_ = 1.0 μL/min. Despite *κ* varying by three orders of magnitude, the volumetric flow rate measurements for all sample liquids remain within the range of *Q*_av_ ± 2σ_*ω*_. This variation of 9% is a great improvement compared to the variation in the previous viscosity-independent pump design, for which mineral oil pumped 526% slower than DI water.

For the new design, we found no correlation between the volumetric flow rate and fluidic properties *κ*, the environmental temperature, the environmental humidity, or the order in which the measurements were performed.

The nitrocellulose paper specifications indicate a large relative variability in the flow rate (10.7%) compared to the one measured with our integrated device (4.4% SD). The flow rate variations in our device are therefore likely caused by variations in the nitrocellulose material.

## Discussions

Removing the dependency of capillary-driven diagnostic tests on sample fluidic properties has the potential to provide more accurate diagnostic assay performance. The flow rates shown in the examples are of relevance for these types of applications, where typically a few microlitres are processed per minute. The flow rate can be adjusted easily if desired. Decreasing the flow rate is accomplished by increasing the fluidic resistance of the flow restrictor, and increasing the flow rate is accomplished by upscaling the device dimensions (see the Supplementary Information). As shown in the Supplementary Information, the novel capillary pump can be straightforwardly adapted to provide the flow conditions needed in a lateral flow test and hence does not create a trade-off for key assay parameters, such as the test sensitivity, specificity, analyte concentration range, or sample volume required.

In this work, the sample channel and pump liquid container were straight circular glass channels. This part of the pump can take almost any form and be made of a large variety of materials. Desired formats could be, e.g., straight or meandering polymer channels with rectangular cross-sections (popular due to the ease of manufacturing) or channels with integrated microarrays or other types of biosensors. Replacing the nitrocellulose with a microstructured absorbent pad with a well-controlled geometry, e.g., a micropillar array used in commercial devices, such as the 4CastChip^TM^, could further reduce the flow variations.

For the integration of the novel pump in lateral flow devices, the following additional design aspects should be considered: the ease-of-use requirements for the activation of the pump, packaging and shelf life of the pump, and integration of a readout region in the sample section of the device. The activation of the pump demonstrator in this work consists of inserting the nitrocellulose insertion region into the pump liquid channel. For a simpler end-user operation, such activation could easily be implemented in the format of a button that mechanically creates a fluidic connection between the pump liquid channel and absorbent pad when pressed. Furthermore, to simplify the fluidic handling required by the end user, pump liquid should be added during device manufacturing. Using a hygroscopic pump liquid instead of water and/or hermetically sealing the pump prior to shipping would prevent the evaporation of the pump liquid during its shelf life.

## Conclusions

This paper introduces a novel capillary pump design with a constant flow that does not depend on the sample viscosity and surface energy. Clinical samples and lab and household liquids for which the filling speed of standard capillaries varies by more than a factor of 10^3^ were capillarily pumped with a constant flow rate with standard deviation less than 5 × 10^−2^.

## Electronic supplementary material

Supplementary Information(DOCX 2931 kb)

Supplementary Figure 1(JPG 1937 kb)

Supplementary Figure 2(JPG 1395 kb)
