# Supplementary material for: Capillary pumping independent of the liquid surface energy and viscosity
Source: Microsyst Nanoeng. 2018 Mar 26;4:2. doi: 10.1038/s41378-018-0002-9 (PMC6220164; doi:10.1038/s41378-018-0002-9)
Supplement: Supplementary file 1 — Supplementary Information(DOCX 2931 kb) [file 41378_2018_2_MOESM1_ESM.docx]

Supplementary file

Capillary pumping independent of liquid surface energy and viscosity

Weijin Guo^1^, Jonas Hansson^1^ & Wouter van der Wijngaart^1, *^

^1^KTH Royal Institute of Technology, Micro and Nanosystems, Osquldas väg 10, 100 44 Stockholm, Sweden

**CORRESPONDENCE** Correspondence and requests for materials should be addressed to Wouter van der Wijngaart (email: [wouter@kth.se](mailto:wouter@kth.se)).

**FLOW BEHAVIOR OF DIFFERENT SAMPLE LIQUIDS IN VISCOSITY INDEPENDENT CAPILLARY PUMPS**

We tested the household lab liquids as sample liquids in the previously reported viscosity independent capillary pump^1^. Figure S1 shows the experimental setup and the measured filling speed. A linear fit of each liquid is also shown in the figure, which’ slope defines the average flow rate. The average flow rate of DI water, ethanol, isopropanol, mineral oil and glycerol are 0.0811 mm/s, 0.0187 mm/s, 0.0216 mm/s, 0.00959 mm/s and 0.0476 mm/s respectively.

**
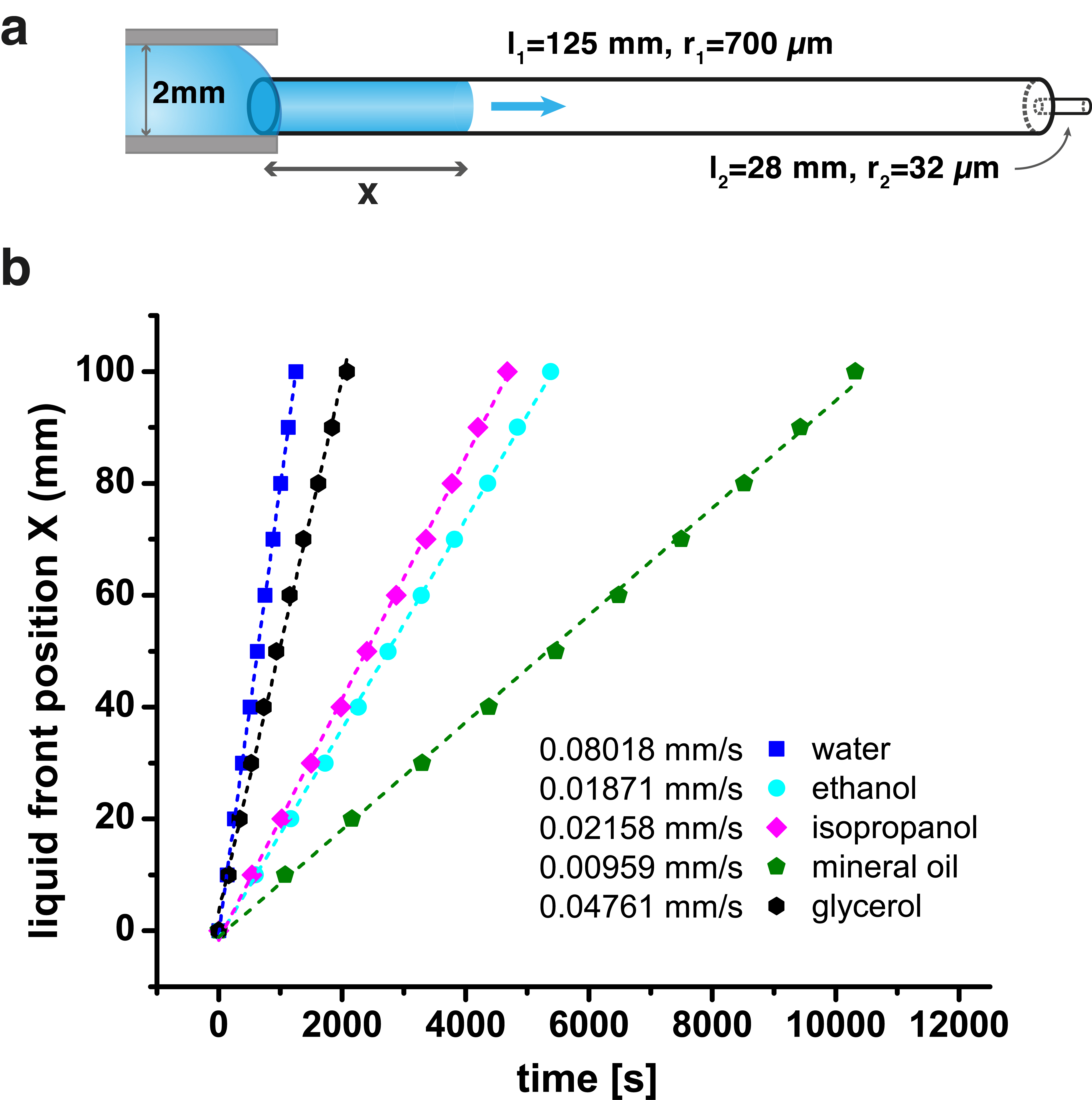
**

**Figure S1** (a) The experimental setup of the viscosity independent capillary pump, adapted from reference [1]. l_1_ and r_1_, and l_2_ and r_2_ indicate the length and radius of the the glass capillary and the polymer air restrictor tube, respectively. (b) The measured filling speed of the different sample liquids, and a linear fit.

**ESTIMATION OF MAXIMUM THEORETICAL FLOW RATE**

In this section we derive an estimate for the maximum flow rate that the novel pump design can deliver. Our pump is designed such that

*P_c,pl_* >> *P_c,pr_* *≈ P_c,sl_ ≈ P_c,sr_ .* (1)

To keep the flow rate constant requires the fluidic resistance of the flow restrictor, *R_R_*, to be much larger than those of the other parts, including the maximum internal flow resistance of the absorbent pad, *RP,max: RP,max << RR*. We define the ratios $f_{P}=\frac{P_{c,pl}}{P_{c,pr}}$ and $f_{R}=\frac{R_{R}}{R_{P,\max}}$ and we can see that the higher the values of *f_P_* and *f_R_*, the more constant flow rate we obtain.

To maximise the volumetric flow rate

$Q\approx\frac{P_{c,pl}}{R_{R}}=\frac{P_{c,pl}}{f_{R}\cdot R_{P,\max}}$ (2) requires maximising *P_c,pl_* while minising *R_P,max_*.

To maximise the capillary pressure and minimise the internal flow resistance of a capillary pump, it must consist of a porous material that is shaped as a section of a sphere with a maximum opening angle and the liquid entrance point at its centre. In a typical 2D realisation, this shape is a pie section of a disc. We define the pore radius *r_P_*, the porous disc thickness *t*, and the opening angle α=2π.

The capillary pressure

$P_{c}=\frac{2\gamma\cos(\theta)}{r_{P}}$, (3) and the internal fluidic resistance for a radial imbibition front position, *R*, equals

$R_{P}=\rho\cdot\frac{-\ln(R)}{\alpha t}$, (4) where ρ is the fluidic resistivity of the porous medium:

$\rho\sim\frac{\mu}{r_{P}^{2}}$. (5)

For a desired pump volume, *V*, and a porous medium with porous fraction, *f_pf_*, the minimum pump radius, *R*, can be determined from $V=\frac{\alpha\cdot t\cdot R^{2}}{2f_{pf}}$ as

$R=\sqrt{\frac{2f_{pf}V}{\alpha t}}$. (6)

Substituting eqs. 3-6 in eq. 2 allows estimating

$Q\approx\frac{P_{c,pl}}{R_{R}}=-\frac{4\gamma\cos(\theta)\alpha t}{f_{R}\cdot\mu\cdot\ln\left(\frac{2f_{pf}V}{\alpha t}\right)}\cdot r{}_{P}$. (7)

For a given pump volume, *V*, the flow rate, *Q*, is maximised when the pore radius, *r_P_*, is maximised. *r_P_* is limited by inequality 1:

$r_{P}=\frac{r_{S}}{f_{P}}$, (8) and *r_S_* is limited by the bond number in the sample section of the pump:

$Bo=\frac{D\cdot g\cdot r_{S}^{2}}{\gamma\cos(\theta)}<1$. (9)

For pump liquid and sample liquid viscosity and density close to those of water and assuming *f_P_* = 10 and *f_R_* = 10, *t = r_S_*, γcos(θ) = 30 mJ/m^2^, f_pf_ = 50%, α = π and a sample volume V = 100 μL, results in *r_S_* ≈ 2 mm, *r_P_* ≈ 200 μm, R ≈ 4 mm, and a maximum volumetric flow rate Q ≈ 1.4 mL/s.

**ADAPTATION OF THE NOVEL PUMP DESIGN FOR LATERAL FLOW TESTING**

In this section, we illustrate a possible approach for adapting a standard lateral flow test with our novel pump design without imposing a trade-off on other key assay parameters, such as test sensitivity, specificity, analyte concentration range or sample volume required.

Consider a standard lateral flow test as illustrated in Figure S2, top.


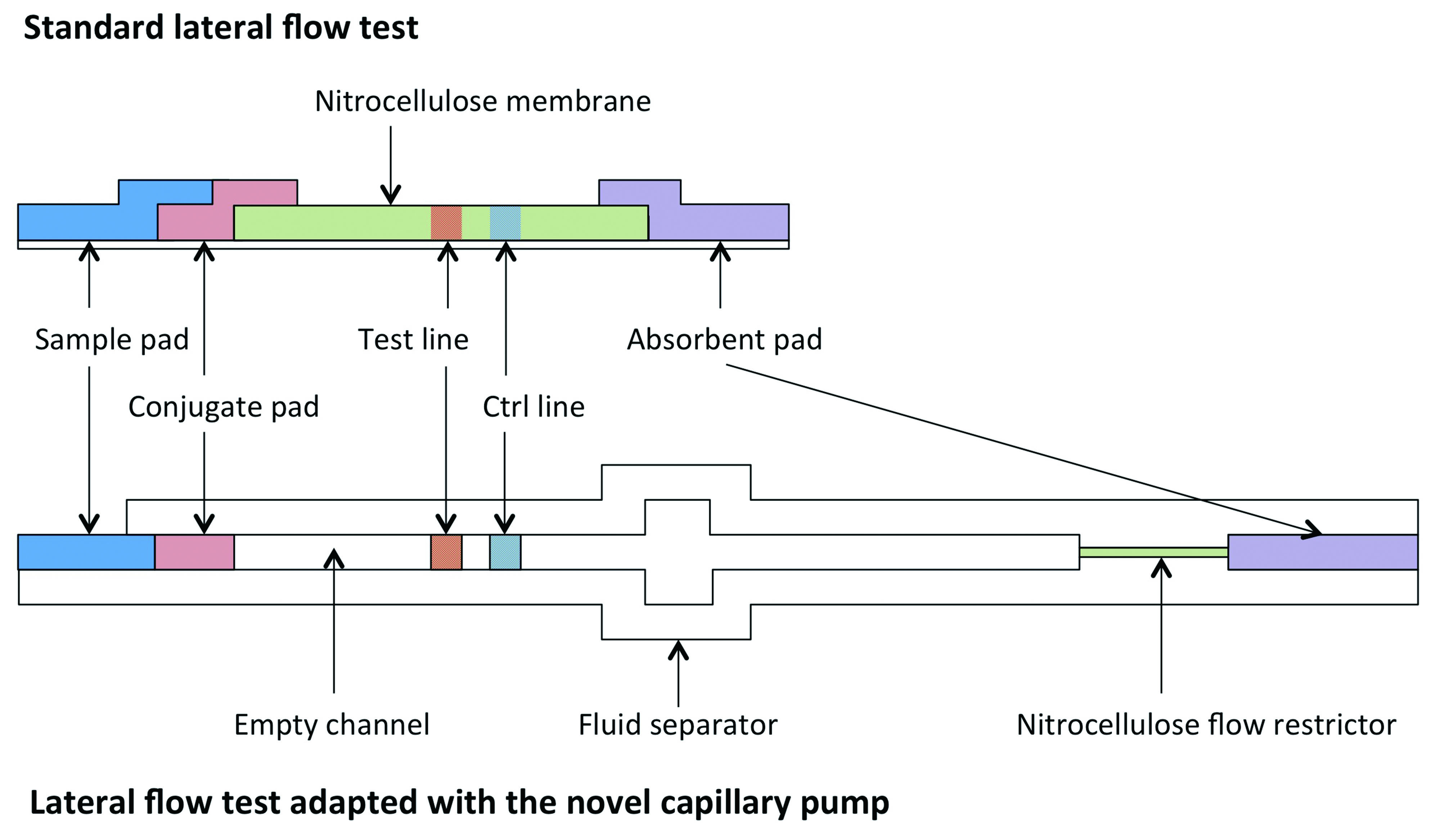


**Figure S2** Cross-sectional illustration of a standard lateral flow test (top) and of a lateral flow test with equal assay conditions, integrated in the novel capillary pump design (bottom).

The fluidic resistance of the sample pad, conjugate pad and absorbent pad of such test are typically designed to be negligible compared to those of the nitrocellulose membrane.

Now consider the integration of this test into the novel capillary pump design, as illustrated in Figure S2, bottom. We incorporate a sample pad, conjugate pad and absorbent pad with the same dimensions as the standard test. The test line and control line, which are strips of nitrocellulose, also retain the same dimensions as those in the standard test. We note here that the flow resistance of the two lines is negligible compared to the flow resistance of the entire nitrocellulose membrane. The flow restrictor consists of a strip of nitrocellulose where the ratio of length over cross-sectional area equals that of the nitrocellulose membrane in the standard test, i.e. they have the same fluidic resistance for a liquid of given viscosity.

We can choose a pump liquid with a viscosity similar to that of the sample.

The flow rate of the standard test will, on average over the full test duration, be equal to that of the novel test, as defined by the capillary pressure of the absorbent pad divided by the fluidic resistance of the nitrocellulose membrane (≈the fluidic resistance of the nitrocellulose flow restrictor).

This results in that the conjugation time will be equal for both designs, and the interaction conditions in the test line and control line will be equal (on average over the full test duration) for both designs.

Hence, the assay conditions for both tests will be basically identical, except for that our novel design removes the test dependency on sample viscosity and surface energy.

**REFERENCES**

1 Guo W, Hansson J, van der Wijngaart W. Capillary Pumping Independent of Liquid Sample Viscosity. *Langmuir* 2016; 32: 12650-12655.
