# Supplementary figures and images for: Capillary pumping independent of the liquid surface energy and viscosity
Source: Microsyst Nanoeng. 2018 Mar 26;4:2. doi: 10.1038/s41378-018-0002-9 (PMC6220164; doi:10.1038/s41378-018-0002-9)

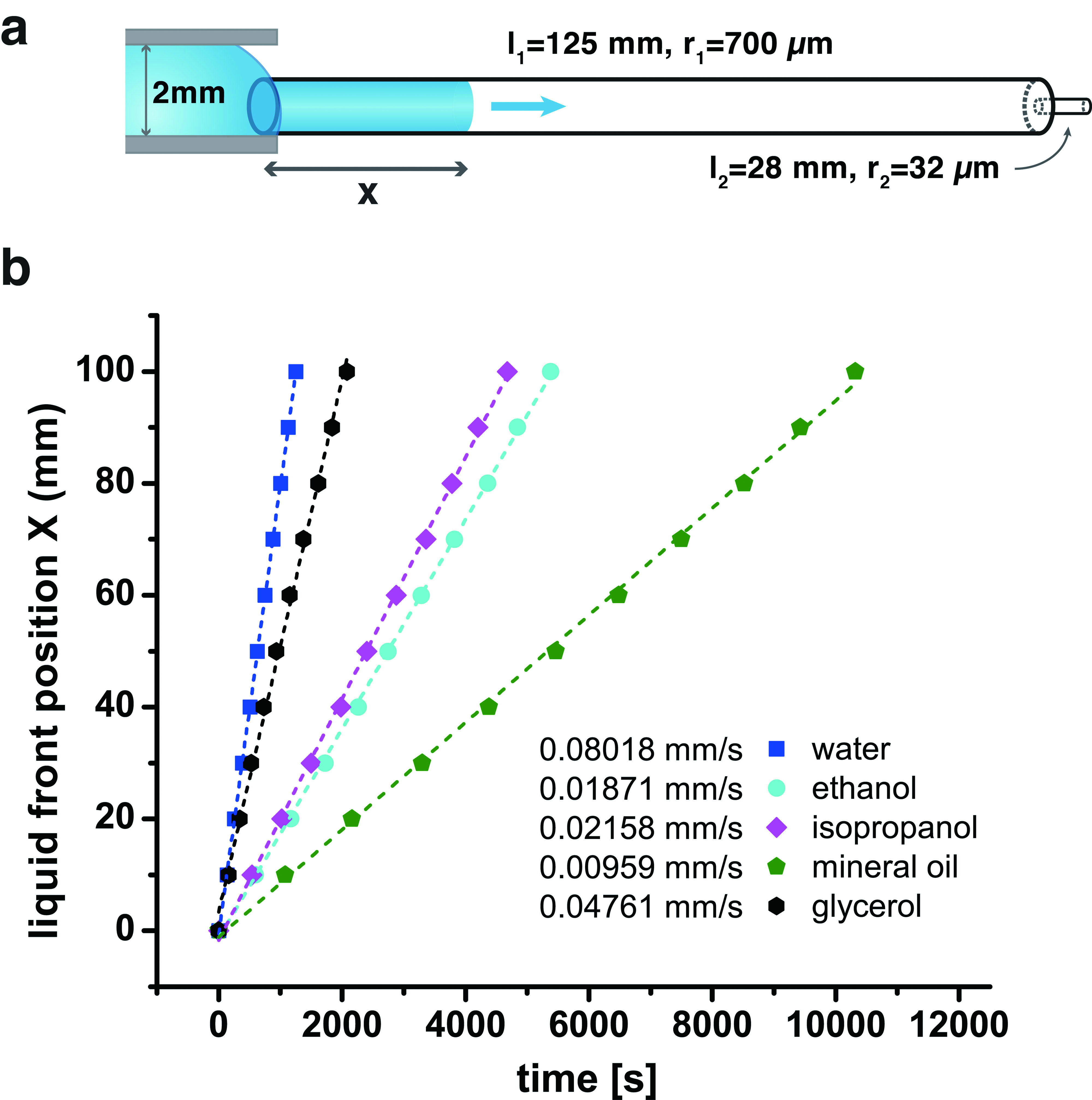

Supplement: Supplementary file 2 — Supplementary Figure 1(JPG 1937 kb) [file 41378_2018_2_MOESM2_ESM.jpg]

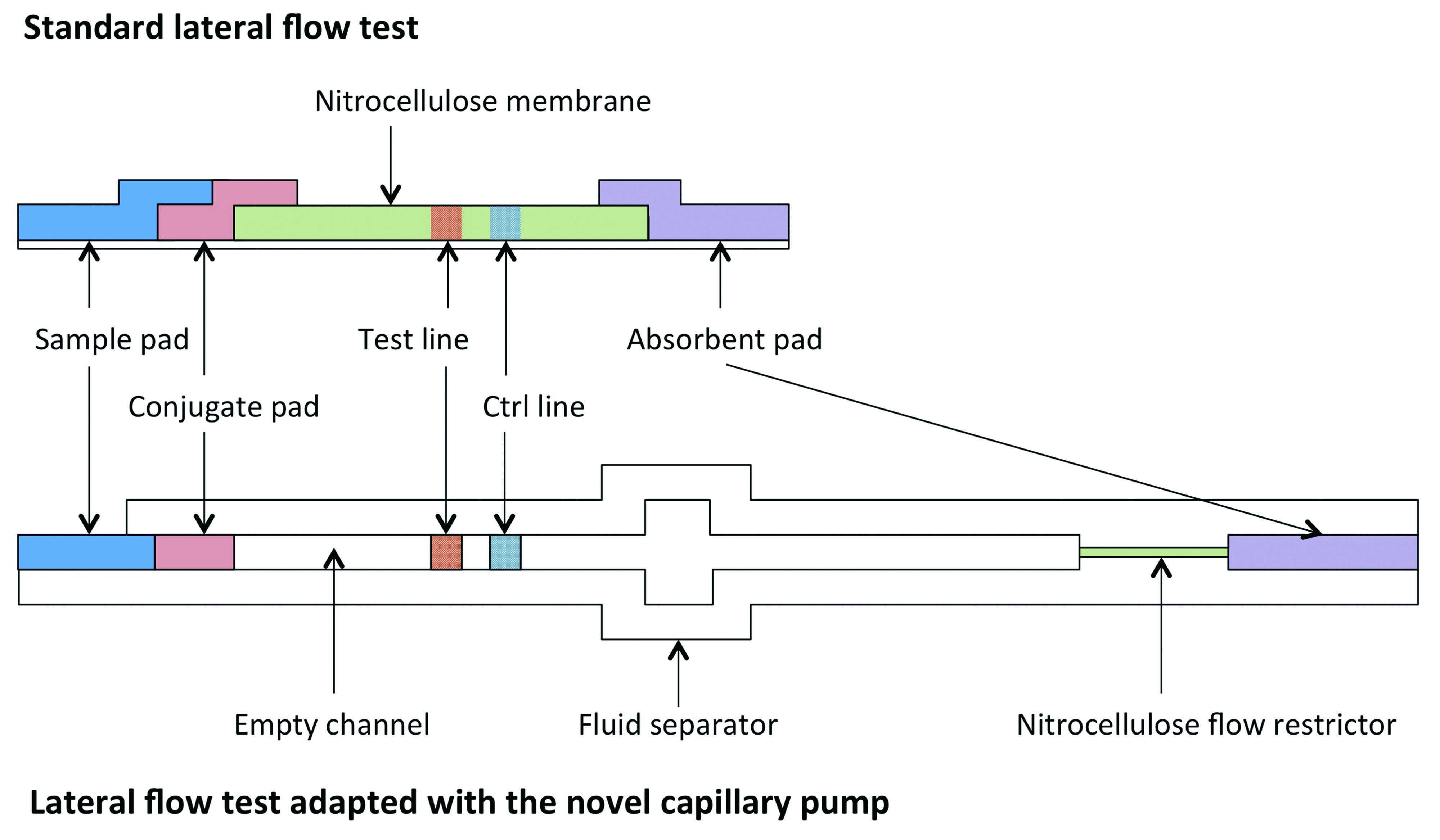

Supplement: Supplementary file 3 — Supplementary Figure 2(JPG 1395 kb) [file 41378_2018_2_MOESM3_ESM.jpg]
